# Chlamydia, gonorrhoea, trichomoniasis and syphilis: global prevalence and incidence estimates, 2016

**DOI:** 10.2471/BLT.18.228486

**Published:** 2019-06-06

**Authors:** Jane Rowley, Stephen Vander Hoorn, Eline Korenromp, Nicola Low, Magnus Unemo, Laith J Abu-Raddad, R Matthew Chico, Alex Smolak, Lori Newman, Sami Gottlieb, Soe Soe Thwin, Nathalie Broutet, Melanie M Taylor

**Affiliations:** a Department of Reproductive Health and Research, World Health Organization, Avenue Appia 20, 1211 Geneva 27, Switzerland.; b School of Mathematics and Statistics, University of Melbourne, Melbourne, Australia.; c Avenir Health, Geneva, Switzerland.; d Institute of Social and Preventive Medicine (ISPM), University of Bern, Bern, Switzerland.; e WHO Collaborating Centre for Gonorrhoea and Other STIs, Örebro University, Örebro, Sweden.; f Department of Healthcare Policy and Research, Weill Cornell Medical College – Qatar, Doha, Qatar.; g Department of Disease Control, London School of Hygiene & Tropical Medicine, London, England.; h Enteric and Sexually Transmitted Infections Branch, National Institute of Allergy and Infectious Diseases, Washington DC, United States of America.

## Abstract

**Objective:**

To generate estimates of the global prevalence and incidence of urogenital infection with chlamydia, gonorrhoea, trichomoniasis and syphilis in women and men, aged 15–49 years, in 2016.

**Methods:**

For chlamydia, gonorrhoea and trichomoniasis, we systematically searched for studies conducted between 2009 and 2016 reporting prevalence. We also consulted regional experts. To generate estimates, we used Bayesian meta-analysis. For syphilis, we aggregated the national estimates generated by using Spectrum-STI.

**Findings:**

For chlamydia, gonorrhoea and/or trichomoniasis, 130 studies were eligible. For syphilis, the Spectrum-STI database contained 978 data points for the same period. The 2016 global prevalence estimates in women were: chlamydia 3.8% (95% uncertainty interval, UI: 3.3–4.5); gonorrhoea 0.9% (95% UI: 0.7–1.1); trichomoniasis 5.3% (95% UI:4.0–7.2); and syphilis 0.5% (95% UI: 0.4–0.6). In men prevalence estimates were: chlamydia 2.7% (95% UI: 1.9–3.7); gonorrhoea 0.7% (95% UI: 0.5–1.1); trichomoniasis 0.6% (95% UI: 0.4–0.9); and syphilis 0.5% (95% UI: 0.4–0.6). Total estimated incident cases were 376.4 million: 127.2 million (95% UI: 95.1–165.9 million) chlamydia cases; 86.9 million (95% UI: 58.6–123.4 million) gonorrhoea cases; 156.0 million (95% UI: 103.4–231.2 million) trichomoniasis cases; and 6.3 million (95% UI: 5.5–7.1 million) syphilis cases.

**Conclusion:**

Global estimates of prevalence and incidence of these four curable sexually transmitted infections remain high. The study highlights the need to expand data collection efforts at country level and provides an initial baseline for monitoring progress of the *World Health Organization global health sector strategy on sexually transmitted infections 2016–202*1.

## Résumé

**Objectif:**

Produire des estimations de la prévalence et de l'incidence mondiales des infections urogénitales dues à la chlamydiose, à la gonorrhée, à la trichomonase et à la syphilis chez les femmes et les hommes de 15 à 49 ans, en 2016.

**Méthodes:**

Pour la chlamydiose, la gonorrhée et la trichomonase, nous avons systématiquement recherché les études menées entre 2009 et 2016 qui s’intéressaient à la prévalence. Nous avons également consulté des experts régionaux. Pour produire des estimations, nous avons eu recours à une méta-analyse bayésienne. Pour la syphilis, nous avons regroupé les estimations nationales obtenues à l'aide de Spectrum-STI.

**Résultats:**

Pour la chlamydiose, la gonorrhée et/ou la trichomonase, 130 études étaient éligibles. Pour la syphilis, la base de données de Spectrum-STI contenait 978 points de données pour la période considérée. Les estimations de la prévalence mondiale en 2016 chez les femmes étaient les suivantes: chlamydiose 3,8% (intervalle d'incertitude de 95%, II: 3,3–4,5); gonorrhée 0,9% (II 95%: 0,7–1,1); trichomonase 5,3% (II 95%: 4,0–7,2); et syphilis 0,5% (II 95%: 0,4–0,6). Chez les hommes, les estimations de la prévalence étaient les suivantes: chlamydiose 2,7% (II 95%: 1,9-3,7); gonorrhée 0,7% (II 95%: 0,5-1,1); trichomonase 0,6% (II 95%: 0,4-0,9); et syphilis 0,5% (II 95%: 0,4–0,6). L'incidence totale estimée était de 376,4 millions de cas: 127,2 millions (II 95%: 95,1–165,9 millions) de cas de chlamydiose; 86,9 millions (II 95%: 58,6-123,4 millions) de cas de gonorrhée; 156,0 millions (II 95%: 103,4-231,2 millions) de cas de trichomonase; et 6,3 millions (II 95%: 5,5–7,1 millions) de cas de syphilis.

**Conclusion:**

Les estimations mondiales de la prévalence et de l'incidence de ces quatre infections sexuellement transmissibles guérissables restent élevées. Cette étude souligne la nécessité d'amplifier les efforts de collecte de données au niveau des pays et offre un point de référence pour suivre la progression de la *Stratégie mondiale du secteur de la santé contre les IST 2016–2021 de l'Organisation mondiale de la Santé.*******

## Resumen

**Objetivo:**

Generar estimaciones de la prevalencia y la incidencia mundiales de la infección urogenital por clamidia, gonorrea, tricomoniasis y sífilis en mujeres y hombres de 15 a 49 años de edad en 2016.

**Métodos:**

Para la clamidia, la gonorrea y la tricomoniasis, se realizaron búsquedas sistemáticas de estudios realizados entre 2009 y 2016 que registrasen la prevalencia. También se consultó a expertos regionales. Para generar estimaciones, se utilizó el metanálisis bayesiano. Para la sífilis, se añadieron las estimaciones nacionales generadas por el uso de Spectrum-STI.

**Resultados:**

Para la clamidia, la gonorrea y/o la tricomoniasis, hubo 130 estudios que cumplían los criterios. Para la sífilis, la base de datos Spectrum-STI contenía 978 puntos de datos para el mismo periodo. Las estimaciones de prevalencia mundial en mujeres en 2016 fueron: clamidia 3,8 % (intervalo de incertidumbre, II, del 95 %: 3,3-4,5); gonorrea 0,9 % (II del 95 %: 0,7-1,1); tricomoniasis 5,3 % (II del 95 %: 4,0-7,2); y sífilis 0,5 % (II del 95 %: 0,4-0,6). Las estimaciones de prevalencia en hombres fueron: clamidia 2,7 % (intervalo de incertidumbre, II, del 95 %: 1,9-3,7); gonorrea 0,7 % (II del 95 %: 0,5-1,1); tricomoniasis 0,6 % (II del 95 %: 0,4-0,9); y sífilis 0,5 % (II del 95 %: 0,4-0,6). El total estimado de casos incidentes fue de 376,4 millones: 127,2 millones (II del 95 %: 95,1-165,9 millones) de casos de clamidia; 86,9 millones (II del 95 %: 58,6-123,4 millones) de casos de gonorrea; 156,0 millones (II del 95 %: 103,4-231,2 millones) de casos de tricomoniasis; y 6,3 millones (II del 95 %: 5,5-7,1 millones) de casos de sífilis.

**Conclusión:**

Las estimaciones mundiales de la prevalencia y la incidencia de estas cuatro enfermedades de transmisión sexual curables siguen siendo elevadas. El estudio destaca la necesidad de ampliar los esfuerzos de recopilación de datos a nivel nacional y proporciona una base inicial para el seguimiento de los progresos de la *Estrategia Mundial del Sector de la Salud de la Organización Mundial de la Salud sobre las ETS entre 2016 y 2021.*

## ملخص

**الغرض:**

وضع تقديرات للانتشار والإصابة العالمية لعدوى الجهاز البولي التناسلي، وأمراض الكلاميديا، والسيلان، وداء المشعرات، والزهري لدى النساء والرجال، الذين تتراوح أعمارهم بين 15 و49 سنة، في عام 2016.

**الطريقة:**

بالنسبة للكلاميديا، والسيلان، وداء المشعرات، فقد قمنا بالبحث بشكل منهجي عن الدراسات التي تمت خلال الفترة من 2009 إلى 2016 والتي توضح مدى الانتشار. كما قمنا باستشارة خبراء إقليميين. ولوضع التقديرات، قمنا باستخدام التحليل التلوي Bayesian. بالنسبة لمرض الزهري، قمنا بتجميع التقديرات الوطنية الناتجة عن استخدام Spectrum-STI.

**النتائج:**

بالنسبة لأمراض الكلاميديا، والسيلان، وداء المشعرات، كانت هناك 130 دراسة مؤهلة. وبالنسبة لمرض الزهري، احتوت قادة بيانات Spectrum-STI على 978 نقطة بيانات لنفس الفترة. كانت تقديرات الانتشار العالمي في عام 2016 في النساء: الكلاميديا 3.8% (فاصل عدم الثقة 95%: 3.3 إلى 4.5)، والسيلان 0.9% (فاصل عدم الثقة 95%: 0.7 إلى 1.1)، وداء المشعرات 5.3% (فاصل عدم الثقة 95%: 4.0 إلى 7.2)؛ والزهري 0.5% (فاصل عدم الثقة 95%: 0.4 إلى 0.6). كانت تقديرات الانتشار في عام 2016 في الرجال: الكلاميديا 2.7% (فاصل عدم الثقة 95%: 1.9 إلى 3.7)، والسيلان 0.7% (فاصل عدم الثقة 95%: 0.5 إلى 1.1)، وداء المشعرات 0.6% (فاصل عدم الثقة 95%: 0.4 إلى 0.9)، والزهري 0.5% (فاصل عدم الثقة 95%: 0.4–0.6). بلغ مجموع حالات الإصابة التقديري 376.4 مليون حالة: 127.2 مليون (فاصل عدم الثقة 95%: 95.1 إلى 165.9 مليون) حالات الكلاميديا؛ 86.9 مليون (فاصل عدم الثقة 95%: 58.6 إلى 123.4 مليون) حالات السيلان؛ 156.0 مليون (فاصل عدم الثقة 95%: 103.4 إلى 231.2 مليون) حالات داء المشعرات؛ 6.3 مليون (فاصل عدم الثقة 95%: 5.5 إلى 7.1 مليون) حالات الزهري.

**الاستنتاج:**

لا تزال التقديرات العالمية عالية لانتشار والإصابة بهذه الأمراض الأربعة المنقولة جنسياً والعلاج منها. تسلط الدراسة الضوء على الحاجة إلى توسيع جهود جمع البيانات على مستوى الدول، وتوفر خط أساس أولي لمراقبة التقدم المحرز في إستراتيجية قطاع الصحة العالمية التابع لمنظمة الصحة العالمية بشأن الأمراض المنقولة جنسياً خلال الفترة من 2016 إلى 2021. ****

## 摘要

**目的:**

旨在估计 2016 年 15-49 岁男女泌尿生殖系统感染衣原体病、淋病、滴虫病和梅毒的全球患病率和发病率。

**方法:**

对于衣原体病、淋病和滴虫病,我们系统搜索了 2009 年至 2016 年间的患病率报告研究。我们还咨询了区域专家。为了生成估计值,我们使用了贝叶斯荟萃分析。对于梅毒,我们汇总分析了使用 Spectrum-STI 生成的全国估计值。

**结果:**

对于衣原体病、淋病和/或滴虫病,符合要求的有 130 项研究。对于梅毒, Spectrum-STI 数据库包含了同一时期的 978 个数据点。2016 年,全球女性患病率估计值为:衣原体病 3.8%(95% 不确定区间,UI:3.3-4.5);淋病 0.9%(95% UI:0.7–1.1);滴虫病 5.3%(95% UI:4.0–7.2)和梅毒 0.5%(95% UI:0.4-0.6)。全球男性患病率估计值为:衣原体病 2.7%(95% UI:1.9-3.7);淋病 0.7%(95% UI:0.5-1.1);滴虫病 0.6%(95% UI:0.4-0.9);梅毒 0.5%(95% UI:0.4-0.6)。预计病例总数为 3.764 亿:1.272 亿(95% UI:9510-16590 万)衣原体病病例;8690 万(95% UI:5860-12340 万)淋病病例;15600 万(95% UI:10340-23120 万)滴虫病病例;630 万(95% UI:550-710 万)梅毒病例。

**结论:**

对这四种可治愈的性传播疾病的患病率和发病率的全球估计值仍然很高。该研究强调了扩大国家级数据收集工作的必要性,并为监测 2016 至 2021 年世卫组织全球卫生部门性传播疾病战略的进展提供了初始基线。****

## Резюме

**Цель:**

Оценить распространенность в мировом масштабе и частоту урогенитальных инфекций, вызываемых хламидией, а также гонореи, трихомониаза и сифилиса у мужчин и женщин в возрасте от 15 до 49 лет по состоянию на 2016 год.

**Методы:**

Для хламидиоза, гонореи и трихомониаза авторы провели систематический поиск исследований, выполненных в период с 2009 по 2016 год, в которых приводились данные по распространенности заболеваний. Авторы также консультировались с международными специалистами. Для оценки использовался байесовский метаанализ. Для исследования сифилиса нами были объединены национальные оценки, созданные с использованием методики Spectrum-STI.

**Результаты:**

Было обнаружено 130 исследований на тему хламидийной инфекции, гонореи и (или) трихомониаза. Что касается сифилиса, база данных Spectrum-STI за тот же период содержала 978 источников данных. По состоянию на 2016 год распространенность изучаемых заболеваний в мире среди женщин составляла: хламидиоз 3,8% (95%-й интервал неопределенности, ИН: 3,3–4,5), гонорея 0,9% (95%-й ИН: 0,7–1,1), трихомониаз 5,3% (95%-й ИН: 4,0–7,2) и сифилис 0,5% (95%-й ИН: 0,4–0,6). У мужчин распространенность хламидиоза составила 2,7% (95%-й ИН: 1,9–3,7), гонореи 0,7% (95%-й ИН: 0,5–1,1), трихомониаза 0,6% (95%-й ИН: 0,4–0,9) и сифилиса 0,5% (95%-й ИН: 0,4–0,6). Общее приблизительное количество случаев заболевания составило 376,4 млн человек: 127,2 млн (95 %-й ИН: 95,1–165,9 млн) случаев хламидийной инфекции, 86,9 млн (95%-й ИН: 58,6–123,4 млн) случаев гонореи, 156,0 млн (95%-й ИН: 103,4–231,2 млн) случаев трихомониаза и 6,3 млн (95%-й ИН: 5,5–7,1 млн) случаев сифилиса.

**Вывод:**

Оценки мировой распространенности и частоты этих четырех излечимых инфекций, передаваемых половым путем (ИППП), остаются высокими. Исследование показывает необходимость предпринимать дальнейшие усилия по сбору данных на уровне каждой страны и может служить источником базовых значений для мониторинга прогресса в исполнении глобальных стратегий ВОЗ в секторе здравоохранения относительно ИППП на период 2016–2021 гг.****

## Introduction

Sexually transmitted infections are among the most common communicable conditions and affect the health and lives of people worldwide. The World Health Organization (WHO) periodically generates estimates to gauge the global burden of four of the most common curable sexually transmitted infections: chlamydia (etiological agent: *Chlamydia trachomatis*), gonorrhoea (*Neisseria gonorrhoeae*), trichomoniasis (*Trichomonas vaginalis*) and syphilis (*Treponema pallidum*).^1^^–^^6^ The estimates provide evidence for programme improvement, monitoring and evaluation.

These sexually transmitted infections cause acute urogenital conditions such as cervicitis, urethritis, vaginitis and genital ulceration, and some of the etiological agents also infect the rectum and pharynx. Chlamydia and gonorrhoea can cause serious short- and long-term complications, including pelvic inflammatory disease, ectopic pregnancy, infertility, chronic pelvic pain and arthritis, and they can be transmitted during pregnancy or delivery. Syphilis can cause neurological, cardiovascular and dermatological disease in adults, and stillbirth, neonatal death, premature delivery or severe disability in infants. All four infections are implicated in increasing the risk of human immunodeficiency virus (HIV) acquisition and transmission.^7^ Moreover, people with sexually transmitted infections often experience stigma, stereotyping, vulnerability, shame and gender-based violence.^8^

In May 2016, the World Health Assembly adopted the *Global health sector strategy on sexually transmitted infections, 2016–2021*.^9^ This strategy includes rapid scale-up of evidence-based interventions and services to end sexually transmitted infections as public health concerns by 2030. The strategy sets targets for reductions in gonorrhoea and syphilis incidence in adults and recommends the establishment of global baseline incidences of sexually transmitted infections by 2018. The primary objectives of this study were to estimate the 2016 global and regional prevalence and incidence of chlamydia, gonorrhoea, trichomoniasis and syphilis in adult women and men.

## Methods

### Prevalence estimation

#### Chlamydia, gonorrhoea and trichomoniasis

We generated estimates for these three infections through systematic reviews using the same methods as for the 2012 estimates.^6^

We searched for articles published between 1 January 2009 and 29 July 2018 in PubMed® without language restrictions. We used PubMed Medical subject heading (MeSH) terms for individual country names combined with: “chlamydia”[MeSH Terms] OR “chlamydia”[All Fields], “gonorrhoea”[All Fields] OR “gonorrhea”[MeSH Terms] OR “gonorrhea”[All Fields], “trichomonas infections”[MeSH Terms] OR (“trichomonas”[All Fields] AND “infections”[All Fields]) OR “trichomonas infections”[All Fields] OR “trichomoniasis”[All Fields]). We also asked WHO regional sexually transmitted infection advisors and other leading experts in the field for additional published and unpublished data. 

To be eligible, studies had to collect most specimens between 2009 and 2016 or be published in 2010 or later if specimen collection dates were not available. Other study inclusion criteria were: sample size of at least 100 individuals; general population (e.g. pregnant women, women at delivery, women attending family planning clinics, men and women selected for participation in demographic and health surveys); and use of an internationally recognized diagnostic test with demonstrated precision using urine, urethral, cervical or vaginal specimens.

To reduce bias in the estimation of general population prevalence, we excluded studies conducted among the following groups: patients seeking care for sexually transmitted infection or urogenital symptoms, women presenting at gynaecology or sexual health clinics with sexually transmitted infection related issues, studies restricted to women with abnormal Papanicolaou test results, remote or indigenous populations, recent immigrant or migrant populations, men who have sex with men and commercial sex workers.

Two investigators independently reviewed all identified studies to verify eligibility. When more than one publication reported on the same population, we retained the publication with the most detailed information. For each included study, we calculated prevalence as the number of individuals with a positive test result divided by the total number tested. We then standardized these values by applying adjustment factors for the accuracy of the laboratory diagnostic test, study location (rural versus urban) and the age of the study population. If the adjustments resulted in a negative value, we replaced the value with 0.1% when doing the meta-analysis. The methods and adjustment factors were identical to those used to generate the 2012 estimates.^6^

We obtained estimates for 10 geographical areas (referred to as estimation regions).^6^ Estimates for high-income North America (Canada and United States of America), were based on the latest published United States estimates that used data from multiple sources.^10^^,^^11^ For the other nine estimation regions, we calculated a summary prevalence estimate by meta-analysis if there were three or more data points.^12^ There were sufficient data to generate an estimate for chlamydia in women in all regions, but not for gonorrhoea or trichomoniasis. For regions with insufficient data for gonorrhoea and trichomoniasis, we assumed that prevalence was a multiple of the prevalence of chlamydia. The infection specific multiples were based on those studies that met the 2016 inclusion criteria (available from the data repository).^13^ For men, when there were insufficient data for meta-analysis, the prevalence of an infection was assumed to be proportional to the prevalence in women. The male-to-female ratios were infection-specific and were set at the same values as in 2012 estimates.^6^

To reflect the contribution of populations at higher risk of infection (e.g. men who have sex with men and commercial sex workers), who are likely to be under-represented in general population samples, we increased prevalence estimates by 10%, as in the 2012 estimates,^6^ for each estimation region, apart from high-income North America.

We performed the meta-analyses using a Bayesian approach with a Markov Chain Monte Carlo algorithm implemented with the software BRrugs in R package (R foundation, Vienna, Austria).^14^ For each infection, the software generated 10 000 samples from the posterior distribution for the expected mean prevalence in each estimation region based on the *β*-binomial model, and used these to calculate the 2.5 and 97.5 uncertainty percentiles.^15^ We calculated global and regional prevalence estimates for each infection by weighting each of the 10 000 samples from estimation regions according to population size, using United Nations population data for women and men aged 15–49 years.^16^ We present results by WHO region, 2016 World Bank income classification^17^ and 2017 sustainable development goal (SDG) region.^18^ All analyses were carried out using R statistical software (R foundation).

#### Syphilis

We based syphilis estimates on the WHO’s published 2016 maternal prevalence estimates.^19^ These estimates were generated by using Spectrum-STI, a statistical trend-fitting model in the publicly available Spectrum suite of health policy planning tools^20^ and country specific data from the global Spectrum-STI syphilis database (available from the corresponding author). As in the 2012 estimation,^6^ we assumed that the prevalence of syphilis in all women 15–49 years of age in each country was the same as in pregnant women. We then increased the estimate by 10% to reflect the contribution of populations at higher risk. The men to women prevalence ratio of syphilis was set at 1.0 and assumed to have a uniform distribution ± 33% around this value, in agreement with data from a recent global meta-analysis of syphilis.^21^

We generated regional and global estimates by weighting the contribution of each country by the number of women and men aged 15–49 years. Regional and global 95% uncertainty intervals (UIs) were generated using the delta method;^22^ uncertainties were assumed to be independent across countries.

### Incidence estimation

We calculated incidence estimates for each infection by dividing prevalence by the average duration of infection for all estimation regions except high-income North America where published estimates were used.^10^^,^^11^ Estimates of the average duration of infection were those used in the 2012 estimation^6^ and assumed to have a uniform distribution of ± 33.3% around the average duration. We calculated uncertainty in incidence for a given region, sex and infection at the national level using the delta method;^22^ uncertainty in the prevalence estimate was multiplied by uncertainty in the estimated duration of infection. Regional and global uncertainty intervals were generated assuming uncertainties were independent across countries.

## Results

### Data availability

#### Chlamydia, gonorrhoea and trichomoniasis

**Fig. 1:**
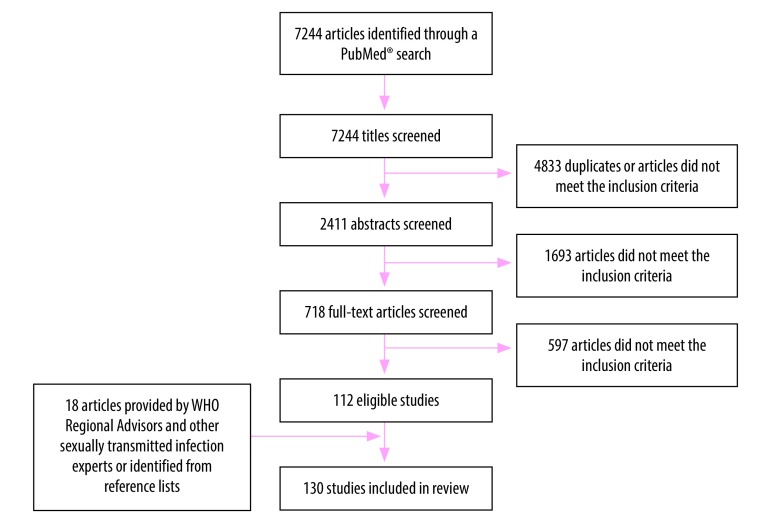
Flowchart of the selection of studies for estimating the prevalence and incidence of chlamydia, gonorrhoea and trichomoniasis, 2016

Of the 7244 articles screened, 112 studies met the inclusion criteria for one or more of the three infections (Fig. 1). We identified an additional 18 studies through expert consultations and reviewing reference lists (Nguyen M et al., Hanoi Medical University, Viet Nam, personal communication, 23 March 2018; El Kettani A et al., National Institute of Hygiene, Morocco, personal communication, 2 May 2016; Galdavadze K et al., Disease Control and Public Health, Republic of Georgia; personal communication, 22 August 2017).^23^^–^^150^ Of these 130 studies, 111 reported data for women only (Table 1; available at: http://www.who.int/bulletin/volumes/96/8/18-228486), three reported data for men only (Table 2; available at: http://www.who.int/bulletin/volumes/96/8/18-228486) and 16 reported data for both women and men (Table 1 and Table 2). Only 34 studies in women and four studies in men provided information on all three infections. The included studies contained 100 data points in women for chlamydia, 64 for gonorrhoea and 69 for trichomoniasis. In men, there were 16 data points for chlamydia, 11 for gonorrhoea and seven for trichomoniasis (Table 3).

WHO: World Health Organization.

Note: This figure does not include studies from North America; the North American estimates were based on published estimates.^10^^,^^11^

**Table 1:** Included studies on chlamydia, gonorrhoea and trichomoniasis prevalence in women, 2009–2016

Study, by WHO region	Country or territory and location	Date of study	Population and age, years	Chlamydia				Gonorrhoea				Trichomoniasis		
				Clinical specimen, test^a^	Sample size	Study prevalence, %		Clinical specimen, test	Sample size	Study prevalence, %		Clinical specimen, test^a^	Sample size	Study prevalence, %
**African Region**														
Wynn et al., 2018^23^	Botswana, Gaborone	Jul 2015–May 2016	ANC clinic attendees, > 18	Genital fluid, amplification test	400	7.8		Genital fluid, amplification test	400	1.3		Genital fluid, amplification test	400	5.3
Ginindza et al., 2017^43^	Eswatini, national^b^	Jun–Jul 2015	Outpatient clinic attendees, 15–49	Genital fluid, amplification test	655	5.8		Genital fluid, amplification test	655	5.3		Genital fluid, amplification test	655	7.8
Eshete et al., 2013^24^	Ethiopia, Jimma Town	Dec 2011–May 2012	ANC clinic attendees, 15–36	NR	NR	NR		NR	NR	NR		Genital fluid, culture	361	5.0
Mulu et al., 2015^25^	Ethiopia, Bahir Dar	May–Nov 2013	ANC clinic attendees, 15–49	NR	NR	NR		Genital fluid, culture and Gram stain^a^	214	0.9		Genital fluid, microscopy	214	1.4
Schönfeld et al., 2018^26^	Ethiopia, Asella	May 2014–Sep 2015	ANC clinic attendees, adults	NR	NR	NR		NR	NR	NR		Genital fluid, point-of-care test^b^	580	5.3
Volker et al., 2017^27^	Ghana, Western region	Oct 2011–Jan 2012	Attendees at a hospital maternity clinic, 14–48	Genital fluid, amplification test	177	1.7		Genital fluid, culture	180	0.0		NR	NR	NR
Jespers et al., 2014^28^	Kenya, Mombasa	2010–2011	Participants in a community survey, 18–35	Genital fluid, amplification test	110	3.6		Genital fluid, amplification test	110	0.9		Genital fluid, culture	110	2.7
Kinuthia et al., 2015^29^	Kenya, Ahero and Bondo districts	May 2011–Jun 2013	ANC clinic attendees, ≥ 14	Genital fluid, amplification test	1276	5.5		Genital fluid, amplification test	1276	2.5		Genital fluid, microscopy	1278	6.3
Drake et al., 2013^30^	Kenya, Western Kenya	Pre-2013	ANC clinic attendees, 14–21	Genital fluid, amplification test	537	4.7		Genital fluid, amplification test	537	1.7		Genital fluid, microscopy	537	5.6
Masese et al., 2017^31^	Kenya, Mombasa	Aug 2014–Mar 2015	Students, 15–24	Urine, amplification test	451	3.5		Urine, amplification test	451	1.6		Urine, amplification test	451	0.7
Masha et al., 2017^32^	Kenya, Kilifi	Jul–Sep 2015	ANC clinic attendees, 18–45	Urine, amplification test	202	14.9		Urine, amplification test	202	1.0		Genital fluid, culture	202	7.4
Nkhoma et al., 2017^33^	Malawi, Mangochi District	Feb 2011–Aug 2012	ANC clinic attendees, ≥ 15	NR	NR	NR		NR	NR	NR		Genital fluid, microscopy	1210	10.5
Olowe et al., 2014^34^	Nigeria, Osogba	Jul–Apr 2012	ANC clinic attendees, adults	NR	NR	NR		NR	NR	NR		Genital fluid, microscopy	100	2.0
Etuketu et al., 2015^35^	Nigeria, Abeokutu	Jun–Jul 2013	ANC clinic attendees, 15–44	NR	NR	NR		NR	NR	NR		Genital fluid, microscopy	300	10.3
Muvunyi et al., 2011^36^	Rwanda, Kigali	Nov 2007–Mar 2010	Controls for infertility study, adults	Genital fluid, amplification test	312	3.8		NR	NR	NR		NR	NR	NR
Franceschi et al., 2016^37^	Rwanda, Kigali	Apr 2013–May 2014	Students, 18–20	Urine, amplification test	912	2.2		NR	NR	NR		NR	NR	NR
Vieira-Baptista et al., 2017^38^	Sao Tome and Principe, Principe	2015	Attendees at a primary health-care clinic, 21–60	Genital fluid, amplification test	100	3.0		Genital fluid, amplification test	100	2.0		Genital fluid, amplification test	100	8.0
Moodley et al., 2015^39^	South Africa, Durban	May 2008–Jun 2010	ANC clinic attendees, adults	Genital fluid, amplification test	1459	17.8		Genital fluid, amplification test	1459	6.4		Genital fluid, amplification test	1459	15.3
Jespers et al., 2014^28^	South Africa, Johannesburg	2010–2011	ANC clinic attendees, adults	Genital fluid, amplification test	109	16.5		Genital fluid, amplification test	109	0.9		Genital fluid, culture	109	4.6
Peters et al., 2014^40^	South Africa, Mopani District	Nov 2011–Feb 2012	Attendees at a primary health-care clinic, 18–49	Genital fluid, amplification test	603	16.1		Genital, amplification test	603	10.1		NR	NR	NR
de Waaij et al., 2017^41^	South Africa, Mopani District	Nov 2011–Feb 2012	Attendees at a primary health-care clinic, 18–49	NR	NR	NR		NR	NR	NR		Genital fluid, amplification test	575	19.7
Francis et al., 2018^42^	South Africa, KwaZulu-Natal	Oct 2016–Jan 2017	Youth people, 15–24	Genital, amplification test	259	11.2		Genital fluid, amplification test	259	1.9		Genital fluid, amplification test	259	4.6
Tchelougou et al., 2013^44^	Togo, Sokodé	Jun 2010–Aug 2011	ANC clinic attendees, adults	NR	NR	NR		NR	NR	NR		Genital fluid, microscopy	302	3.6
Donders et al., 2016^45^	Uganda, Kampala	Pre-2015	Outpatient clinic attendees, adult	Genital fluid, amplification test	360	1.4		Genital fluid, amplification test	360	1.7		Genital fluid, amplification test	360	6.7
Rutherford et al., 2014^46^	Uganda, Kampala	Sep 2008–Apr 2009	Students, 19–25	Genital fluid, amplification test	280	2.5		Genital fluid, amplification test	280	1.1		Genital fluid, culture	247	0.8
de Walque et al., 2012^47^	United Republic of Tanzania, Kilombero and Ulanga Districts	Feb–Apr 2009	Participants in HIV prevention trial, 18–30	Genital fluid, amplification test	1204	2.7		Genital fluid, amplification test	1204	1.4		Genital fluid, amplification test	1204	16.2
Chiduo et al., 2012^48^	United Republic of Tanzania, Tanga	May 2009–Oct 2010	ANC clinic attendees, 18–44	Genital fluid, amplification test	185	1.6		Genital fluid, culture and Gram stain	185	1.6		Genital fluid, microscopy	185	11.4
Hokororo et al., 2015^49^	United Republic of Tanzania, Mwanza	Apr–Dec 2012	ANC clinic attendees, 14–20	Urine, amplification test	403	11.4		Urine, amplification test	403	6.7		Genital fluid, microscopy	403	13.4
Lazenby et al., 2014^50^	United Republic of Tanzania, Arusha District	Pre-2014	Participants for cervical cancer screening, 30–60	Genital fluid, amplification test	324	0.0		Genital fluid, amplification test	324	0.0		Genital fluid, amplification test	297	10.4
Maufi et al., 2018^51^	United Republic of Tanzania, Mwanza	Nov 2014–Apr 2015	ANC clinic attendees, 17–46	NR	NR	NR		NR	NR	NR		Genital fluid, microscopy	365	10.4
Chaponda et al., 2016^52^	Zambia, Nchelenge District	Nov 2013–Apr 2014	ANC clinic attendees, adults	Genital fluid, amplification test	1083	5.2		Genital fluid, amplification test	1083	3.1		Genital fluid, amplification test	1083	24.8
Stephen et al., 2017^53^	Zimbabwe, Harare	Jan 2012–Apr 2012	ANC clinic attendees, > 18	Genital fluid, amplification test	242	5.8		NR	NR	NR		NR	NR	NR
**Region of the Americas**														
Touzon et al., 2014^54^	Argentina, Buenos Aires	Jan 2010–Dec 2012	ANC clinic attendees, adults	NR	NR	NR		NR	NR	NR		Genital fluid, culture	1238	1.8
Testardini et al., 2016^55^	Argentina, Buenos Aires	Apr 2010–Aug 2011	ANC clinic attendees, adults	NR	NR	NR		NR	NR	NR		Genital fluid, amplification test	386	5.2
Mucci et al., 2016^56^	Argentina, Buenos Aires	Aug 2012–Jan 2013	ANC clinic attendees, 10–42	NR	NR	NR		Genital fluid, culture	210	0.5		Genital fluid, microscopy	210	1.4
Department of Public Health 2018^57^	Bahamas, national	2016	ANC clinic attendees, adults	Urine, amplification test	2504	12.0		Urine, amplification test	2504	2.0		NR	NR	NR
Magalhaes et al., 2015^58^	Brazil, Rio Grande do Norte State	2008–2012	Participants for cervical cancer screening, 25–60	Genital fluid, amplification test	1134	10.9		NR	NR	NR		NR	NR	NR
Miranda et al., 2014^59^	Brazil, national	Mar–Nov 2009	ANC clinic attendees, 15–24	NR	NR	NR		NR	NR	NR		Genital fluid, amplification test	299	7.7
Pinto et al., 2011^60^	Brazil, national	Mar–Nov 2009	ANC clinic attendees, 15–24	Urine, amplification test	2071	9.8		Urine, amplification test	2071	1.0		NR	NR	NR
Ferreira et al., 2015^61^	Brazil, Belem and Para	2009–2011	ANC clinic attendees, < 19	Genital fluid, amplification test	168	16.7		NR	NR	NR		Genital fluid, culture	168	3.0
Piazzetta et al., 2011^62^	Brazil, Curitiba	Pre-2011	Sexually active youth people, 16–23	Urine, amplification test	335	10.7		Urine, amplification test	335	1.5		NR	NR	NR
Silveira MF et al., 2017^63^	Brazil, Pelotas	Dec 2011–May 2013	Attendees at a hospital maternity clinic, 18–24	Genital fluid, amplification test	562	12.3		NR	NR	NR		NR	NR	NR
Mesenburg et al., 2013^64^	Brazil, Pelotas	Dec 2011–Jan 2013	ANC clinic attendees, < 30	Genital fluid, amplification test	361	15.0		NR	NR	NR		NR	NR	NR
Gatti et al., 2017^65^	Brazil, Rio Grande	Jan 2012–Jan 2015	ANC clinic attendees, adults	NR	NR	NR		NR	NR	NR		Genital fluid, amplification test	204	5.9
Marconi et al., 2015^66^	Brazil, Botucatu	Sep 2012–Jan 2013	Participants for cervical cancer screening, 14–54	NR	NR	NR		NR	NR	NR		Genital fluid, microscopy	1519	1.4
Neves et al., 2016^67^	Brazil, Manaus	Oct 2012–Dec 2013	Attendees at a primary health-care clinic, 14–25	Genital fluid, amplification test	1169	13.1		NR	NR	NR		NR	NR	NR
Zamboni et al., 2016^68^	Brazil, Santiago	Mar 2013–Mar 2014	Outpatient clinic attendees, 15–24	Genital fluid, amplification test	181	5.5		NR	NR	NR		NR	NR	NR
Melo et al., 2016^69^	Brazil, Region of La Araucanía	2013–2014	Participants for cervical cancer screening, 18–24	Genital fluid, amplification test	151	11.3		NR	NR	NR		NR	NR	NR
Glehn et al., 2016^70^	Brazil, Federal District	Nov 2014–Mar 2015	Attendees at a primary health-care clinic, 18–49	NR	NR	NR		NR	NR	NR		Genital fluid, culture	193	15.5
Ovalle et al., 2012^71^	Chile, Santiago	Apr 2010–Oct 2010	ANC clinic attendees, adults	Genital fluid, amplification test	255	5.9		Genital fluid, culture	255	0.0		Genital fluid, culture	255	2.4
Huneeus et al., 2018^72^	Chile, Santiago	2012–2014	Sexually active youth people, < 25	Genital fluid, amplification test	171	8.8		Genital fluid, amplification test	171	0.6		Genital fluid, amplification test	171	0.0
Villaseca et al., 2015^73^	Chile, Santiago	Jun 2013–Dec 2013	Attendees at a family health clinic, 15–54	NR	NR	NR		NR	NR	NR		Genital fluid, amplification test	101	3.0
Stella et al., 2011^74^	Colombia, rural Medellin	2009–2010	Students, 15–18	NR	NR	NR		Genital fluid, culture	262	0.0		NR	NR	NR
Paredes et al., 2015^75^	Colombia, Sabana Centro province	2011	Students, 14–19	Urine, amplification test	436	3.2		Urine, amplification test	436	0.2		NR	NR	NR
Giraldo-Ospina et al., 2015^76^	Colombia, Dosquebradas	Jun 2012–Aug 2013	ANC clinic attendees, 15–47	Genital fluid, amplification test	101	0.0		Genital fluid, culture	101	2.0		NR	NR	NR
Ceron et al., 2014^77^	Colombia, Bogota	Aug–Dec. 2013	ANC clinic attendees, 15–40	Genital fluid, amplification test	226	5.3		Genital fluid, amplification test	226	0.0		NR	NR	NR
Jobe et al., 2014^78^	Haiti, Jérémie	Oct 2012	Attendees at a primary health-care clinic, 16–75	Genital fluid, amplification test	199	11.6		Genital fluid, amplification test	199	4.0		Genital fluid, amplification test	199	19.6
Jobe et al., 2014^78^	Haiti, Jérémie	Oct 2012	Attendees at a primary health-care clinic, 19–78	Genital fluid, amplification test	104	1.9		Genital fluid, amplification test	104	1.0		Genital fluid, amplification test	104	13.5
Scheildell et al., 2018^79^	Haiti, Gressier	Aug–Oct 2013	ANC clinic attendees, adults	Urine, amplification test	200	8.0		Urine, amplification test	200	3.0		Urine, amplification test	200	20.5
Bristow et al., 2017^80^	Haiti, Port-au-Prince	Oct 2015–Jan 2016	ANC clinic attendees, > 18	Genital fluid, amplification test	300	14.0		Genital fluid, amplification test	300	2.7		Genital fluid, amplification test	300	27.7
Conde-Ferráez et al., 2017^81^	Mexico, Merida	Aug 2010–Jan 2011	ANC clinic attendees, adults	Genital fluid, amplification test	121	8.3		NR	NR	NR		NR	NR	NR
López-Monteon et al., 2013^82^	Mexico, central Veracruz	Jun–Jul 2012	Attendees at a primary health-care clinic, 14–50	NR	NR	NR		NR	NR	NR		Urine, amplification test	158	19.0
Magana-Contreras et al., 2015^83^	Mexico, Villahermosa	Jan 2013–Nov 2014	Participants for cervical cancer screening, 16–74	Genital fluid, amplification test	201	1.5		NR	NR	NR		NR	NR	NR
Casillas-Vega et al., 2017^84^	Mexico, Jalisco	Sep 2013–Aug 2014	ANC clinic attendees, adults	Genital fluid, amplification test	287	10.8		NR	NR	NR		NR	NR	NR
Cabeza et al., 2015^85^	Peru, Lima	Dec 2012–Jan 2013	ANC clinic attendees, ≥ 16	Genital fluid, amplification test	600	10.0		NR	NR	NR		NR	NR	NR
van der Helm et al., 2013^86^	Suriname, Paramaribo	Mar 2008–Jul 2010	Attendees at a family planning clinic, adults	Genital fluid, amplification test	819	9.5		NR	NR	NR		NR	NR	NR
van der Helm et al., 2012^87^	Suriname, Paramaribo	Jul 2009–Feb 2010	Attendees at a family planning clinic, > 18	Genital fluid, amplification test	753	9.2		NR	NR	NR		NR	NR	NR
**South-East Asia Region**														
Franceschi et al., 2016^37^	Bhutan, Thimpu and Paro	Sep 2013	Students in an HPV vaccination study, 18–20	Urine, amplification test	973	3.4		NR	NR	NR		NR	NR	NR
Vidwan et al., 2012^88^	India, Vellore	Apr 2009–Jan 2010	ANC clinic attendees, 18–45	Genital fluid, amplification test	784	0.1		NR	NR	NR		NR	NR	NR
Vijaya Mn et al., 2013^89^	India, rural Bangalore	Oct 2010–Sep 2012	Attendees at an obstetrics and gynaecology clinic, 25–46	NR	NR	NR		NR	NR	NR		Genital fluid, culture	750	2.1
Kojima et al., 2018^90^	India, Mysore district	May 2011–Jun 2014	ANC clinic attendees, young women	Genital fluid, amplification test	213	0.5		Genital fluid, amplification test	213	0.9		Genital fluid, amplification test	213	6.1
Shah et al., 2014^91^	India, Baroda	May 2011–Aug 2012	ANC clinic attendees, 20–35	NR	NR	NR		NR	NR	NR		Genital fluid, microscopy	233	3.4
Krishnan et al., 2018^92^	India, Udupi district	Aug 2013–May 2015	Community members, 18–65	Urine, amplification test	811	0.2		Urine, amplification test	811	0.0		NR	NR	NR
Ani & Darmayani 2017^93^	Indonesia, Bali	Apr 2010	ANC clinic attendees, adults	NR	NR	NR		NR	NR	NR		Genital fluid, culture	376	7.4
Banneheke et al., 2013^94^	Sri Lanka, Colombo district	2007–2009	Participants in diagnostic test study, 16–45	NR	NR	NR		NR	NR	NR		Genital fluid, microscopy	601	2.8
**European Region**														
Farr et al., 2016^95^	Austria, Vienna	Jan 2005–Jan 2015	ANC clinic attendees, adults	NR	NR	NR		NR	NR	NR		Genital fluid, DNA probe-based assay^c^	3763	0.8
Ljubin-Sternak et al., 2017^96^	Croatia, Zagreb	Mar 2014–Feb 2015	Attendees at an obstetrics and gynaecology clinic, adults	Genital fluid, amplification test	8665	1.7		NR	NR	NR		NR	NR	NR
Peuchant et al., 2015^97^	France, Bordeaux	Jan–Jun 2011	ANC clinic attendees, 18–44	Genital fluid, amplification test	1004	2.5		Genital fluid, amplification test	1004	0.0		NR	NR	NR
Peuchant et al., 2015^97^	France, Bordeaux	Sep 2012–Feb 2013	ANC clinic attendees, < 25	Genital fluid, amplification test	112	7.1		Genital fluid, amplification test	112	1.8		NR	NR	NR
Galdavadze et al., personal communication 2012	Georgia, Tbilisi	Jul 2011–Mar 2012	ANC clinic attendees, 14–44	Urine, amplification test	300	5.0		Urine, amplification test	300	0.3		NR	NR	NR
Ikonomidis et al., 2015^98^	Greece, Thessaly state	Feb 2012–Nov 2015	Attendees at a urology and gynaecology clinic, adults	Genital fluid, amplification test	130	0.8		NR	NR	NR		NR	NR	NR
O'Higgins et al., 2017^99^	Ireland, Dublin	Dec 2011–Dec 2013	ANC clinic attendees, 16–25	Genital fluid, amplification test	2687	4.9		NR	NR	NR		NR	NR	NR
Hassan et al., 2016^100^	Ireland, Dublin	Jul 2014–Jan 2015	Participants for cervical cancer screening, 25–40	Genital fluid, amplification test	236	3.0		Genital fluid, amplification test	236	0.0		NR	NR	NR
Bianchi et al., 2016^101^	Italy, Milan	Dec 2008–Dec 2012	HPV vaccinated young women, 18–23	Genital fluid, amplification test	591	4.9		NR	NR	NR		NR	NR	NR
Seraceni et al., 2016^102^	Italy, north-eastern	Jan 2009–Dec 2014	Participants for cervical cancer screening, adults	Genital fluid, amplification test	921	0.0		NR	NR	NR		NR	NR	NR
Panatto et al., 2015^103^	Italy, Turin, Milan and Genoa	Jan–Jun 2010	Women attending gynaecologic routine check-ups, 16–26	Genital fluid, amplification test	566	5.8		NR	NR	NR		NR	NR	NR
Foschi et al., 2016^104^	Italy, Bologna	Jan 2011–May 2014	Attendees at an obstetrics and gynaecology clinic, routine, > 14	Genital fluid, amplification test	3072	3.4		NR	NR	NR		NR	NR	NR
Matteelli et al., 2016^105^	Italy, Brescia	Nov 2012–Mar 2013	Sexually active students, ≥ 18	Urine, amplification test	1297	1.9		Urine, amplification test	1297	0.0		NR	NR	NR
Camporiondo et al., 2016^106^	Italy, Rome	Mar 2013	Healthy women attending screening, 34–60	Genital fluid, amplification test	309	0.0		Genital fluid, amplification test	309	0.0		Genital fluid, amplification test	309	1.3
Leli et al., 2016^107^	Italy, Perugia	Jan–Oct 2015	Outpatient clinic attendees, adults	NR	NR	NR		NR	NR	NR		Genital fluid, amplification test	1487	1.3
Gravningen et al., 2013^108^	Norway, Finnmark	2009	Sexually active students, 15–20	Urine, amplification test	607	6.8		NR	NR	NR		NR	NR	NR
Silva et al., 2013^109^	Portugal, Porto	Pre-2013	Students, 14–30	Genital fluid, amplification test	432	6.9		NR	NR	NR		NR	NR	NR
Babinská et al., 2017^110^	Slovakia, eastern parts	2011	Community members, adults	Urine, amplification test	511	3.5		NR	NR	NR		NR	NR	NR
Fernández-Benítez et al., 2013^111^	Spain, Laviana and Asturias	Nov 2010–Dec 2011	Sexually active youth people, 15–24	Urine, amplification test	277	4.0		NR	NR	NR		NR	NR	NR
Pineiro et al., 2016^112^	Spain, Basque Autonomous Community	Jan 2011–Dec 2014	Attendees at a hospital maternity clinic, 14–54	Urine, amplification test	11 687	1.0		Urine, amplification test	11 687	0.0		NR	NR	NR
Field et al., 2018^113^	United Kingdom, national	Sep 2010–Aug 2012	Sexually active adults, 16–44	NR	NR	NR		NR	NR	NR		Urine, amplification test	2559	0.3
Sonnenberg et al., 2013^114^	United Kingdom, national	Sep 2010–Aug 2012	Sexually active adults, 16–44	Urine, amplification test	2665	2.3		Urine, amplification test	2665	0.1		NR	NR	NR
**Eastern Mediterranean Region**														
Nada et al., 2015^115^	Egypt, Cairo	Jan–Nov 2014	Controls for infertility study, adult	Genital fluid, amplification test	100	2.0		NR	NR	NR		NR	NR	NR
Hassanzadeh et al., 2013^116^	Iran (Islamic Republic of), Shiraz	2009–2011	ANC clinic attendees, adults	NR	NR	NR		Genital fluid, amplification test	1100	1.2		NR	NR	NR
Hamid et al., 2011^117^	Iran (Islamic Republic of), Zanjan province	Apr 2009	Attendees at an obstetrics and gynaecology clinic, 15–45	NR	NR	NR		Genital fluid, culture	328	0.9		NR	NR	NR
Nourian et al., 2013^118^	Iran (Islamic Republic of), Zanjan	Jul 2009–Jun 2010	ANC clinic attendees, adults	NR	NR	NR		NR	NR	NR		Genital fluid, culture	1000	3.3
Rasti et al., 2011^119^	Iran (Islamic Republic of), Kashan	Pre-2010	ANC clinic attendees, adults	NR	NR	NR		NR	NR	NR		Genital fluid, culture	450	0.4
Dehgan Marvast et al., 2017^120^	Iran (Islamic Republic of), Yazd	May–Sep 2010	ANC clinic attendees, 16–39	Urine, amplification test	250	0.0		NR	NR	NR		NR	NR	NR
Ahmadi et al., 2016^121^	Iran (Islamic Republic of), Sanandaj	Aug 2012–Jan 2013	Controls for spontaneous abortion study, 19–42	Genital fluid, amplification test	109	11.9		NR	NR	NR		NR	NR	NR
Arbabi et al., 2014^122^	Iran (Islamic Republic of), Kashan	Oct 2012–Aug 2013	Attendees at a public health unit, 16–60	NR	NR	NR		NR	NR	NR		Genital fluid, culture	970	2.3
Hasanabad et al., 2013^123^	Iran (Islamic Republic of), Sabzevar	Pre-2013	ANC clinic attendees, adolescents	Urine, amplification test	399	12.3		Urine, amplification test	399	1.3		NR	NR	NR
Mousavi et al., 2014^124^	Iran (Islamic Republic of), Sanandai	Feb–May 2013	Controls for infertility study, 14–40	Genital fluid, amplification test	104	5.8		NR	NR	NR		NR	NR	NR
Nateghi Rostami et al., 2016^125^	Iran (Islamic Republic of), Qom	May 2013–Apr 2014	Attendees at an obstetrics and gynaecology clinic, 18–50	Genital fluid, amplification test	518	7.1		NR	NR	NR		NR	NR	NR
Marashi et al., 2014^126^	Iran (Islamic Republic of), not specified	Pre-2014	Controls for infertility study, 20–40	Genital fluid, amplification test	200	6.5		NR	NR	NR		NR	NR	NR
Joolayi et al., 2017^127^	Iran (Islamic Republic of), Ahvaz	Aug 2016–Jan 2017	Controls for infertility study, 18–49	Genital fluid, amplification test	125	1.6		NR	NR	NR		NR	NR	NR
El Kettani et al., personal communication, 2016	Morocco, Rabat, Salé, Agadir and Fes	Oct 2011–Dec 2011	Attendees at a family planning clinic, 18–49	Genital fluid, amplification test	537	3.0		Genital fluid, amplification test	537	0.4		Genital, culture	537	5.6
El Kettani et al., personal communication, 2016	Morocco, Rabat, Salé, Agadir and Fes	Dec 2011–Jan 2012	ANC clinic attendees, 18–49	Genital fluid, amplification test	252	3.6		Genital fluid, amplification test	252	0.8		Genital fluid, culture	252	5.2
Kamel 2013^128^	Saudi Arabia, Jazan	Jul 2011–Jun 2012	Controls for infertility study, 18–40	Genital fluid, culture	100	4.0		NR	NR	NR		NR	NR	NR
**Western Pacific Region**														
Wen 2013^129^	China, Wuhu	2010	Sexually active adults, adults	NR	NR	NR		NR	NR	NR		Genital fluid, microscopy	2010	6.6
Lu et al., 2013^130^	China, Shenzhen	2011–2012	Attendees at an obstetrics and gynaecology clinic, adults	Genital fluid, amplification test	7892	5.4		NR	NR	NR		NR	NR	NR
Xia et al., 2015^131^	China, east, 16 cities	Jan–Dec 2011	Attendees at an hospital maternity clinic, adults	Genital fluid, culture	108 268	1.5		NR	NR	NR		NR	NR	NR
Zhang et al., 2017^132^	China, Shaanxi province	Jun 2012–Jan 2013	ANC clinic attendees, adults	Genital fluid, amplification test	500	3.4		NR	NR	NR		NR	NR	NR
Zhang et al., 2017^133^	China, Beijing	Mar–Oct 2014	Attendees at an obstetrics and gynaecology clinic, 20–70	Genital fluid, amplification test	953	2.2		Genital fluid, amplification test	953	0.0		Genital fluid, microscopy	953	1.7
Imai et al., 2015^134^	Japan, Miyazaki	Oct 2011–Feb 2012	Students, > 18	Urine, amplification test	1183	3.7		NR	NR	NR		NR	NR	NR
Suzuki et al., 2015^135^	Japan, national	Oct 2013–Mar 2014	ANC clinic attendees, adults	Genital fluid, amplification test	250 571	2.3		NR	NR	NR		NR	NR	NR
Ministry of Health 2017^136^	Mongolia, national	2016	ANC clinic attendees, adults	NR	NR	NR		Genital fluid, culture	69 278	0.5		NR	NR	NR
Corsenac et al., 2015^137^	New Caledonia, national	Aug–Dec 2012	Attendees at a primary health-care clinic, 18–49	Urine, amplification test	376	10.1		Urine, amplification test	376	3.5		NR	NR	NR
Unger et al., 2015^138^	Papua New Guinea, Madang	Nov 2009–Aug 2012	ANC clinic attendees, ≥ 16	Genital fluid, amplification test	674	4.5		Genital fluid, amplification test	674	8.2		Genital fluid, amplification test	674	21.8
Wangnapi et al., 2015^139^	Papua New Guinea, Madang	Feb 2011–Apr 2012	ANC clinic attendees, 16–39	Genital fluid, amplification test	362	11.0		Genital fluid, amplification test	362	9.7		Genital fluid, amplification test	362	21.3
Vallely et al., 2017^140^	Papua New Guinea, four provinces	Dec 2011–Jan 2015	ANC clinic attendees, 18–59	Genital fluid, amplification test	765	22.9		Genital fluid, amplification test	765	14.2		Genital fluid, amplification test	765	22.4
Vallely et al., 2017^140^	Papua New Guinea, four provinces	Dec 2011–Jan 2015	Participants for cervical cancer screening, 18–59	Genital fluid, amplification test	614	7.5		Genital fluid, amplification test	614	8.0		Genital fluid, amplification test	614	15.0
Badman et al., 2016^141^	Papua New Guinea, Milne Bay	Aug–Dec 2014	ANC clinic attendees, > 18	Genital fluid, amplification test	125	20.0		Genital fluid, amplification test	125	11.2		Genital fluid, amplification test	125	37.6
Hahn et al., 2014^142^	Republic of Korea, Seoul	Mar 2010–Apr 2011	ANC clinic attendees, adults	Genital fluid, amplification test	455	2.2		Genital fluid, amplification test	455	0.4		Genital fluid, amplification test	455	0.0
Choe et al., 2012^143^	Republic of Korea, Seoul	Mar–Dec 2010	Attendees at a health examination clinic, 20–59	Urine, amplification test	805	3.2		Urine, amplification test	805	0.2		NR	NR	NR
Kim et al., 2011^144^	Republic of Korea, Uijeongbu	Jul–Dec 2010	Attendees at a check-up clinic, 20–60	Genital fluid, amplification test	279	3.9		Genital fluid, amplification test	279	0.4		Genital fluid, amplification test	279	2.5
Kim et al., 2014^145^	Republic of Korea, Seoul	Jan–Oct 2012	Attendees at a health examination clinic, 25–81	Genital fluid, amplification test	405	1.2		Genital fluid, amplification test	405	0.0		Genital fluid, amplification test	405	0.2
Marks et al., 2015^146^	Solomon Islands, Honiara	Aug 2014	Attendees at a primary health-care clinic, 16–49	Genital fluid, amplification test	296	20.3		Genital fluid, amplification test	296	5.1		NR	NR	NR
Ton Nu et al., 2015^147^	Viet Nam, Hue	Sep 2010–Jun 2012	Attendees at a family planning clinic, adults	NR	NR	NR		NR	NR	NR		Genital fluid, microscopy	534	0.7
Nguyen et al., personal communication, 2017	Viet Nam, Hanoi	2016–2017	ANC clinic attendees, > 18	Genital fluid, amplification test	490	6.9		Genital fluid, amplification test	490	0.0		Genital fluid, amplification test	490	0.8

ANC: antenatal care; DNA: deoxyribonucleic acid; HIV: human immunodeficiency virus; NR: not reported; WHO: World Health Organization.

^a^ Studies that reported using both culture and Gram stain were assumed to have the same sensitivity and specificity values as culture.

^b^ The study used an immunochromatographic capillary-flow enzyme immunoassay and we assumed a sensitivity of 50% and specificity of 99%.

^c^ The study used a nonamplified, nucleic acid probe-based test system and we assumed the same specific and sensitivity values as for a nucleic acid amplification test.

**Table 2:** Included studies on chlamydia, gonorrhoea and trichomoniasis prevalence in men, 2009–2016

Study, by WHO region	Country or territory and location	Date of study	Population and age, years	Chlamydia				Gonorrhoea				Trichomoniasis		
				Clinical specimen, test^a^	Sample size	Study prevalence, %		Clinical specimen, test^a^	Sample size	Study prevalence, %		Clinical specimen, test^a^	Sample size	Study prevalence, %
**African Region**														
Francis et al., 2018^42^	South Africa, KwaZulu-Natal	Oct 2016–Jan 2017	Community members, 15–24	Urine, amplification test	188	5.3		Urine, amplification test	188	1.6		Urine, amplification test	188	0.5
Rutherford et al., 2014^46^	Uganda, Kampala	Sep 2008–Apr 2009	Students, 19–25	Urine, amplification test	360	0.8		Urine, amplification test	360	0.0		NR	NR	NR
de Walque et al., 2012^47^	United Republic of Tanzania, Kilombero and Ulanga districts	Feb–April 2009	Participants in HIV prevention trial, 18–30	Urine, amplification test	1195	1.7		Urine: amplification test	1195	0.4		Urine, amplification test	1195	8.5
**Region of the Americas**														
Huneeus et al., 2018^72^	Chile, Santiago	2012–2014	Sexually active students, ≤ 24	Urine, amplification test	115	8.7		Urine, amplification test	115	0.0		Urine, amplification test	115	0.0
Paredes et al., 2015^75^	Colombia, Sabana Centro province	2011	Students, 14–19	Urine, amplification test	536	1.1		Urine, amplification test	536	0.0		NR	NR	NR
**South-East Asia Region**														
Jatapai et al., 2013^148^	Thailand, national	Nov 2008–May 2009	Military recruits, 17–29	Urine, amplification test	2123	7.9		Urine, amplification test	2123	0.9		NR	NR	NR
**European Region**														
Sviben et al., 2015^149^	Croatia, Zagreb	Pre-2014	Controls in case-control study, 18–66	NR	NR	NR		NR	NR	NR		Urine, amplification test	200	2.0
Ikonomidis et al., 2015^98^	Greece, Thessaly State	Feb 2012–Nov 2015	Attendees at urology and gynaecology clinic, adult	Genital, amplification test	171	0.6		NR	NR	NR		NR	NR	NR
Matteelli et al., 2016^105^	Italy, Brescia	Nov 2012–Mar 2013	Sexually active students, > 18	Urine, amplification test	762	1.4		Urine, amplification test	762	0.0		NR	NR	NR
Gravningen et al., 2013^108^	Norway, Finnmark	2009	Sexually active youth, 15–20	Urine, amplification test	505	3.4		NR	NR	NR		NR	NR	NR
Babinská et al., 2017^110^	Slovakia, eastern parts	2011	Community members, adult	Urine, amplification test	344	2.0		NR	NR	NR		NR	NR	NR
Fernández-Benítez et al., 2013^111^	Spain, Laviana and Asturias	Nov 2010–Dec 2011	Sexually active youth, 15–24	Urine, amplification test	210	4.3		NR	NR	NR		NR	NR	NR
Field et al., 2018^113^	United Kingdom, national	Sep 2010–Aug 2012	Sexually active adults, 16–44	NR	NR	NR		NR	NR	NR		Urine, amplification test	1827	0.0
Sonnenberg et al., 2013^114^	United Kingdom, national	Sep 2010–Aug 2012	Sexually active adults, 16–44	Urine, amplification test	1885	1.9		Urine, amplification test	1885	0.1		NR	NR	NR
**Eastern Mediterranean Region**														
Arbabi et al., 2014^122^	Iran (Islamic Republic of), Kashan	Oct 2012–Aug 2013	Attendees at a public health unit, 16–60	NR	NR	NR		NR	NR	NR		Genital fluid, culture	233	0.9
Yeganeh et al., 2013^150^	Iran (Islamic Republic of), Tehran	Pre-2013	Urology clinic attendees, 18–50	Urine, amplification test	100	4.0		NR	NR	NR		NR	NR	NR
**Western Pacific Region**														
Corsenac et al., 2015^137^	New Caledonia, national	Aug–Dec 2012	Attendees at a primary health-care clinic, 18–49	Urine, amplification test	232	7.8		Urine, amplification test	232	3.4		NR	NR	NR
Choe et al., 2012^143^	Republic of Korea, Seoul	Mar–Dec 2010	Attendees at a health examination clinic, 20–59	Urine, amplification test	807	7.9		Urine, amplification test	807	0.6		NR	NR	NR
Kim et al., 2011^144^	Republic of Korea, Uijeongbu	Jul–Dec 2010	Attendees at a check-up clinic, 20–60	Urine, amplification test	430	6.7		Urine, amplification test	430	0.5		Urine, amplification test	430	0.2

HIV: human immunodeficiency virus; NR: not reported; WHO: World Health Organization.

^a^ Tests were either nucleic acid amplification test or culture.

**Table 3:** Number of data points that met the study inclusion criteria for the WHO 2016 prevalence estimates of chlamydia, gonorrhoea and trichomoniasis

Estimation region	No. of countries, territories and areas	Chlamydia						Gonorrhoea						Trichomoniasis				
		Women			Men			Women			Men			Women			Men	
		No. of data points	No. of countries		No. of data points	No. of countries		No. of data points	No. of countries		No. of data points	No. of countries		No. of data points	No. of countries		No. of data points	No. of countries
Central, eastern and western sub-Saharan Africa	41	16	7		2	2		15	7		2	2		21	9		1	1
Southern sub-Saharan Africa	6	7	4		1	1		6	3		1	1		6	3		1	1
Andean, central, southern and tropical Latin America and Caribbean	42	25	8		2	2		14	6		2	2		16	5		1	1
High-income North America	2	NA	NA		NA	NA		NA	NA		NA	NA		NA	NA		NA	NA
North Africa and Middle East	20	11	4		1	1		5	2		0	0		5	2		1	1
Australasia and high-income Asia Pacific	6	6	2		2	1		4	1		2	1		3	1		1	1
Western, central and eastern Europe and central Asia	54	19	11		6	6		9	7		2	2		4	3		2	2
Oceania	14	7	3		1	1		7	3		1	1		5	1		0	0
South Asia	5	4	2		0	0		2	1		0	0		3	1		0	0
East Asia and south-east Asia	15	5	2		1	1		2	2		1	1		6	4		0	0
**Total**	**205**	**100**	**43**		**16**	**15**		**64**	**32**		**11**	**10**		**69**	**29**		**7**	**7**

NA: not applicable; WHO: World Health Organization.

Note: Eight of the 112 studies with data for women had two separate data points (e.g. for different population groups).

For women, a total of 43 (21.0%) of 205 countries, territories and areas had one or more data points for chlamydia, 32 (15.6%) for gonorrhoea and 29 (14.1%) for trichomoniasis. For men, only 15 (7.3%) countries, territories and areas had one or more data points for chlamydia, 10 (4.9%) for gonorrhoea and 7 (3.4%) for trichomoniasis. For women there were sufficient data to generate summary estimates for chlamydia for the nine estimation regions, but not for gonorrhoea or trichomoniasis (Table 4).

**Table 4:** Approach used to generate 2016 regional estimates for chlamydia, gonorrhoea and trichomoniasis

Estimation region	Women				Men		
	Chlamydia	Gonorrhoea	Trichomoniasis		Chlamydia	Gonorrhoea	Trichomoniasis
Central, eastern and western sub-Saharan Africa	Meta-analysis	Meta-analysis	Meta-analysis		Global male-to-female ratio	Global male-to-female ratio	Global male-to-female ratio
Southern sub-Saharan Africa	Meta-analysis	Meta-analysis	Meta-analysis		Global male-to-female ratio	Global male-to-female ratio	Global male-to-female ratio
Andean, central, southern and tropical Latin America and Caribbean	Meta-analysis	Meta-analysis	Meta-analysis		Special case^a^	Global male-to-female ratio	Global male-to-female ratio
High-income North America^b^	United States estimate for 2012	United States estimate for 2008	United States estimate for 2008		United States estimate for 2012	United States estimate for 2008	United States estimate for 2008
North Africa and Middle East	Meta-analysis	Meta-analysis	Meta-analysis		Global male-to-female ratio	Global male-to-female ratio	Global male-to-female ratio
Australasia and high-income Asia Pacific	Meta-analysis	Gonorrhoea to chlamydia ratio	Trichomoniasis to chlamydia ratio		Global male-to-female ratio	Global male-to-female ratio	Global male-to-female ratio
Western, central and eastern Europe and central Asia	Meta-analysis	Meta-analysis	Trichomoniasis to chlamydia ratio		Meta-Analysis	Global male-to-female ratio	Global male-to-female ratio
Oceania	Meta-analysis	Meta-analysis	Meta-Analysis		Global male-to-female ratio	Global male-to-female ratio	Global male-to-female ratio
South Asia	Meta-analysis	Gonorrhoea to chlamydia ratio	Trichomoniasis to chlamydia ratio^c^		Global male-to-female ratio	Global male-to-female ratio	Global male-to-female ratio
East Asia and south-east Asia	Meta-analysis	Gonorrhoea to chlamydia ratio^d^	Meta-analysis		Global male-to-female ratio	Global male-to-female ratio	Global male-to-female ratio

^a^ In consultation with advisors on sexual transmitted infections for the World Health Organization (WHO) Region of the Americas, we decided to use the midpoint between the 2016 estimate generated by applying the global male-to-female ratio (7.5%) and the 2012 estimate for the region (2.1%). We deemed the former to be too high and the latter too low.

^b^ Following discussions with the United States Centers for Disease Control and Prevention, we based our estimates on the latest published United States national estimates^21^^,^^22^ and assumed they remained constant over time and that estimates for 15–39-year-old people could be extrapolated to the 15–49-year age range. We did not apply the adjustments used for other Regions in the WHO estimates process. The figures for the United States were also applied to Canada.

^c^ The estimate based on the three available data points was over 4%, considerably higher than the 2012 estimate. Following discussions with regional experts we decided not to use this estimate, but instead to use the trichomoniasis to chlamydia ratio for low and lower middle-income countries, territories and areas.

^d^ This estimation region is made up of countries from East Asia and South East Asia. We used the higher and upper-middle income gonorrhoea to chlamydia ratio for East Asia and the low and lower-middle income ratio for South East Asia.”

#### Syphilis

As of 2 May 2018, the Spectrum-STI Database contained 1576 data points from surveys conducted since 1990, including 978 from January 2009 to December 2016.^151^ In total, 181 (88.3%) of 205 countries, territories and areas had sufficient data to generate a Spectrum STI estimate for 2016. For the remaining 24 countries, territories and areas, we used the median value of the countries with data for the relevant WHO region as the 2016 estimate.

### Prevalence and incidence estimates

Table 5 shows prevalence estimates for the WHO regions for 2016. Based on prevalence data from 2009 to 2016, the estimated pooled global prevalence of chlamydia in 15–49-year-old women was 3.8% (95% UI: 3.3–4.5) and in men 2.7% (95% UI: 1.9–3.7), with regional values ranging from 1.5 to 7.0% in women and 1.2 to 4.0% in men. For gonorrhoea, the global estimate was 0.9% (95% UI: 0.7–1.1) in women and 0.7% (95% UI: 0.5–1.1) in men, with regional values in women ranging from 0.3 to 1.9% and from 0.3 to 1.6% in men. The estimates for trichomoniasis were 5.3% (95% UI: 4.0–7.2) in women and 0.6% (95% UI: 0.4–0.9) in men, with regional values ranging from 1.6 to 11.7% in women and from 0.2 to 1.3% in men. For syphilis, the global estimate in both men and women was 0.5% (95% UI: 0.4–0.6) with regional values ranging from 0.1 to 1.6%. The WHO African Region had the highest prevalence for chlamydia in men, gonorrhoea in women and men, trichomoniasis in women and syphilis in men and women. The WHO Region of the Americas had the highest prevalence of chlamydia in women and of trichomoniasis in men.

**Table 5:** Comparison of 2012 and 2016 WHO regional prevalence estimates of chlamydia, gonorrhoea, trichomoniasis and syphilis

WHO Region, by sex	Estimated prevalence, % (95% UI)											
	Chlamydia			Gonorrhoea			Trichomoniasis			Syphilis		
	2012	2016		2012	2016		2012	2016		2012	2012 (updated)	2016
**Women**												
African Region	3.7 (2.7–5.2)	5.0 (3.8–6.6)		1.7 (1.2–2.6)	1.9 (1.3–2.7)		11.5 (9.0–14.6)	11.7 (8.6–15.6)		1.8 (1.4–2.5)	1.7 (1.5–1.9)	1.6 (1.2–2.0)
Region of the Americas	7.6 (6.7–8.7)	7.0 (5.8–8.3)		0.8 (0.5–1.1)	0.9 (0.6–1.5)		7.7 (4.3–13.1)	7.7 (5.1–11.5)		0.4 (0.4–0.5)	0.7 (0.6–0.7)	0.9 (0.7–1.1)
South-East Asia Region	1.8 (1.4–2.2)	1.5 (1.0–2.5)		0.4 (0.2–0.5)	0.7 (0.4–1.2)		1.8 (1.1–2.7)	2.5 (1.3–4.9)		0.4 (0.3–0.4)	0.4 (0.2–0.5)	0.2 (0.1–0.4)
European Region	2.2 (1.6–2.9)	3.2 (2.5–4.2)		0.3 (0.2–0.5)	0.3 (0.1–0.6)		1.0 (0.8–1.3)	1.6 (1.1–2.3)		0.2 (0.1–0.4)	0.1 (0.1–0.1)	0.1 (0.0–0.4)
Eastern Mediterranean Region	3.5 (2.4–5.0)	3.8 (2.6–5.4)		0.5 (0.3–0.7)	0.7 (0.5–1.1)		5.9 (4.5–8.0)	4.7 (3.3–6.7)		0.5 (0.4–0.9)	0.6 (0.5–0.8)	0.7 (0.4–1.0)
Western Pacific Region	6.2 (5.1–7.5)	4.3 (3.0–5.9)		1.2 (0.8–1.7)	0.9 (0.5–1.3)		5.5 (3.3–8.9)	5.6 (2.7–10.8)		0.2 (0.2–0.3)	0.3 (0.2–0.4)	0.2 (0.1–0.4)
Global total	4.2 (3.7–4.7)	3.8 (3.3–4.5)		0.8 (0.6–1.0)	0.9 (0.7–1.1)		5.0 (4.0–6.4)	5.3 (4.0–7.2)		0.4 (0.4–0.6)	0.5 (0.5–0.6)	0.5 (0.5–0.6)
**Men**												
African Region	2.5 (1.7–3.6)	4.0 (2.4–6.1)		0.5 (0.3–0.9)	1.6 (0.9–2.6)		1.2 (0.7–1.7)	1.2 (0.7–1.8)		1.8 (1.1–2.8)	1.7 (1.4–2.0)	1.6 (1.2–2.0)
Region of the Americas	1.8 (1.3–2.6)	3.7 (2.1–5.5)		0.7 (0.4–1.0)	0.8 (0.4–1.3)		1.3 (0.9–2.0)	1.3 (0.9–1.8)		0.4 (0.3–0.6)	0.7 (0.5–0.8)	0.9 (0.7–1.2)
South-East Asia Region	1.3 (0.9–1.8)	1.2 (0.6–2.1)		0.5 (0.3–0.8)	0.6 (0.3–1.1)		0.2 (0.1–0.3)	0.2 (0.1–0.5)		0.4 (0.2–0.5)	0.4 (0.2–0.5)	0.2 (0.2–0.4)
European Region	1.5 (0.9–2.6)	2.2 (1.5–3.0)		0.3 (0.2–0.5)	0.3 (0.1–0.5)		0.1 (0.1–0.2)	0.2 (0.1–0.3)		0.2 (0.1–0.4)	0.1 (0.1–0.2)	0.1 (0.0–0.3)
Eastern Mediterranean Region	2.7 (1.6–4.3)	3.0 (1.7–4.8)		0.4 (0.2–0.6)	0.6 (0.3–1.0)		0.6 (0.4–0.9)	0.5 (0.3–0.7)		0.5 (0.3–0.9)	0.6 (0.5–0.8)	0.7 (0.4–1.0)
Western Pacific Region	5.2 (3.4–7.2)	3.4 (2.0–5.3)		1.0 (0.6–1.7)	0.7 (0.4–1.2)		0.6 (0.3–1.0)	0.6 (0.2–1.1)		0.2 (0.2–0.3)	0.3 (0.2–0.4)	0.2 (0.1–0.4)
Global total	2.7 (2.0–3.6)	2.7 (1.9–3.7)		0.6 (0.4–0.9)	0.7 (0.5–1.1)		0.6 (0.4–0.8)	0.6 (0.4–0.9)		0.5 (0.3–0.7)	0.5 (0.5–0.6)	0.5 (0.4–0.6)

UI: uncertainty interval; WHO: World Health Organization.

Notes: The 2012 estimates are from Newman et al., 2015.^6^ For syphilis both the WHO estimate for 2012 and the 2018 updated 2012 estimate using Spectrum STI are shown.^19^ For chlamydia, gonorrhoea and trichomoniasis, the study inclusion window for 2016 was samples collected between 2009 and 2016, and for 2012, between 2005 and 2012.

These prevalence estimates correspond to the totals of 124.3 million cases of chlamydia, 30.6 million cases of gonorrhoea, 110.4 million cases of trichomoniasis and 19.9 million cases of syphilis (available from the data repository).^13^

**Fig. 2:**
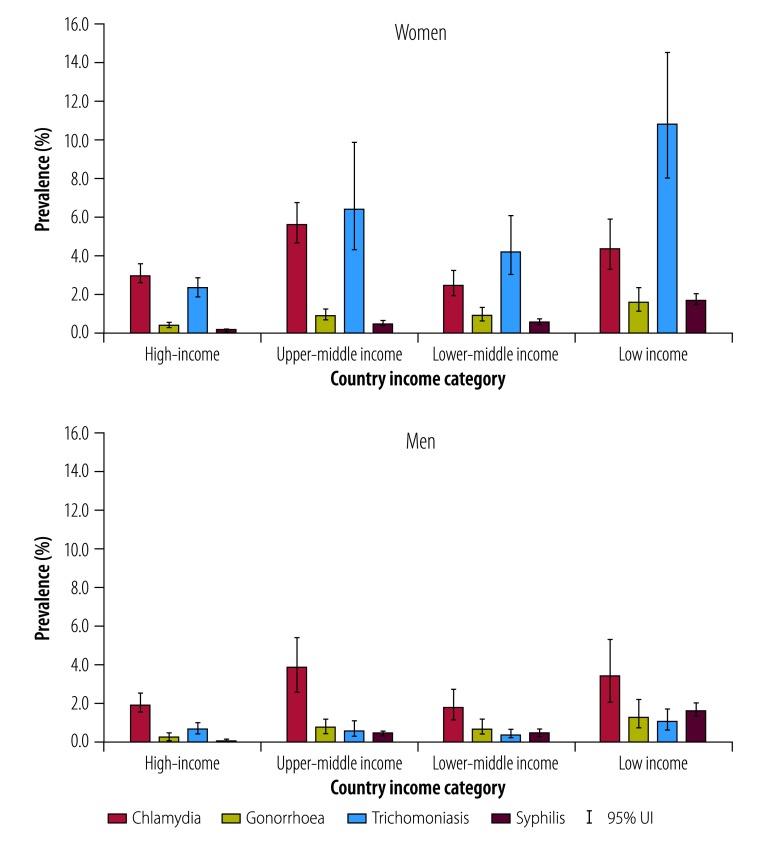
Prevalence estimates of chlamydia, gonorrhoea, trichomoniasis and syphilis in adults, by World Bank classification, 2016

Using the World Bank classification, high-income countries, territories and areas had the lowest estimated prevalence, and low-income countries, territories and areas had the highest prevalence of gonorrhoea, trichomoniasis and syphilis. For chlamydia, estimated prevalence was highest in upper-middle income countries, territories and areas (Fig. 2). The SDG grouping showed the highest prevalence of all four sexually transmitted infections in Oceania region, that is, Pacific island nations excluding Australia and New Zealand (available from the data repository).^13^

UI: uncertainty interval.

Notes: We defined adults as 15–49 years of age and used year 2016 income classification from the World Bank.^17^

**Fig. 3:**
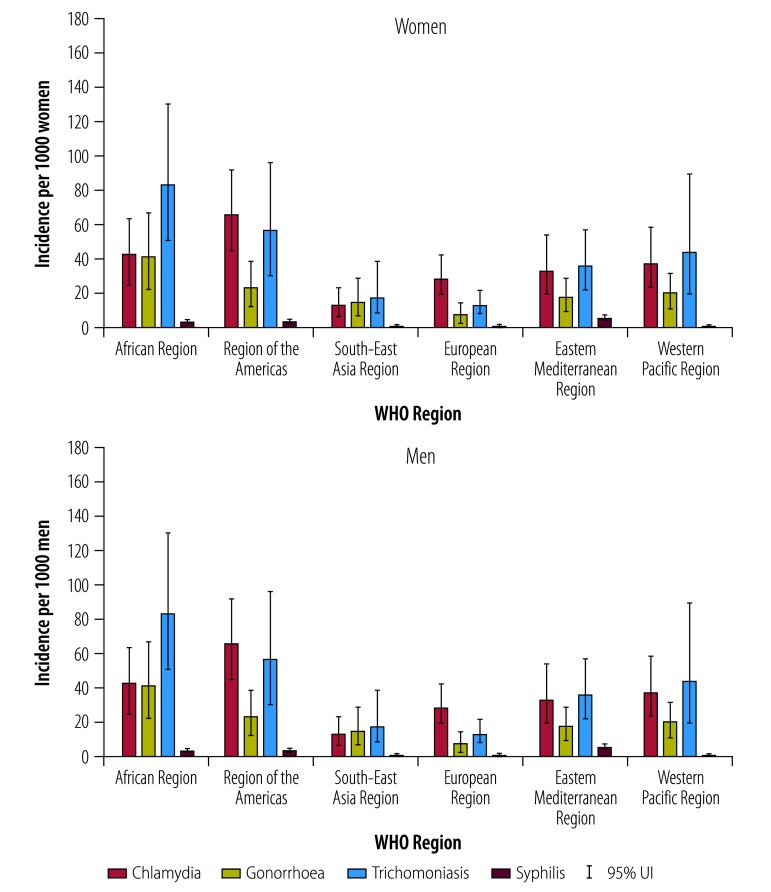
Incidence rate estimates for chlamydia, gonorrhoea, trichomoniasis and syphilis in adults, by WHO Region, 2016

We estimated the global incidence rate for chlamydia in 2016 to be 34 cases per 1000 women (95% UI: 25–45) and 33 per 1000 men (95% UI: 21–48); for gonorrhoea 20 per 1000 women (95% UI: 14–28) and 26 per 1000 men (95% UI: 15–41); for trichomoniasis 40 per 1000 women (95% UI: 27–58) and 42 per 1000 men (95% UI: 23–69); and for syphilis 1.7 per 1000 women (95% UI: 1.4–2.0) and 1.6 per 1000 men (95% UI: 1.3–1.9; Fig. 3). The WHO Region of the Americas had the highest incidence rate for chlamydia and syphilis in both women and men, while the WHO African Region had the highest incidence rates for gonorrhoea and trichomoniasis in women and men. Incidence rates by income category and SDG regions are available from the data repository.^13^

UI: uncertainty interval, WHO: World Health Organization.

Note: We defined adults as 15–49 years of age.

These incidence rates translate globally into 127.2 million (95% UI: 95.1–165.9) new chlamydia cases, 86.9 million (95% UI: 58.6–123.4 million) gonorrhoea cases, 156.0 million (95% UI: 103.4–231.2 million) trichomoniasis cases and 6.3 million (95% UI: 5.5–7.1 million) syphilis cases in women and men aged 15–49 years in 2016. Together, the four infections accounted for 376.4 million new infections in 15–49-year-old people in 2016. Approximately 13.5% (50.8 million) of these infections occurred in low-income countries, territories and areas, 31.4% (118.1 million) in lower middle income, 47.1% (177.3 million) in upper-middle income and 8.0% (30.1 million) in high-income (available from the data repository).^13^

### Comparison of estimates

Comparing the 2012 estimates with the estimates presented here shows that more data points were available in women for the 2016 estimates. The number increased from 69 to 100 for chlamydia, 50 to 64 for gonorrhoea and 44 to 69 for trichomoniasis. For men, the number of data points fell from 21 to 16 for chlamydia and from 12 to 11 for gonorrhoea, but increased from one to seven for trichomoniasis. The period of eligibility for both estimates was eight years with an overlap of four years (2009 to 2012); in women 27 data points were included in both estimates for chlamydia, 18 for gonorrhoea and 20 for trichomoniasis. In men, these overlaps were six, five and one, respectively.

Table 5 compares the 2012 and 2016 prevalence estimates for the four infections. For syphilis, two estimates are presented for 2012, the published estimate^6^ and the 2012 estimate generated using Spectrum STI and the latest Spectrum data set.^19^ For all infections in both women and men, the 2016 global prevalence estimate was within the 95% UI for 2012. At the regional level, the 95% UIs for prevalence overlapped for all four infections in both men and women, apart from gonorrhoea in men in the WHO African Region which was higher in 2016 than in 2012.

## Discussion

We estimated a global total of 376.4 million new curable urogenital infections with chlamydia, gonorrhoea, trichomoniasis and syphilis in 15–49-year-old women and men in 2016. This estimate corresponds to an average of just over 1 million new infections each day. The number of individuals infected, however, is smaller as repeat infections and co-infections are common.^152^

The estimates of prevalence and incidence in 2016 were similar to those in 2012, both globally and by region, showing that sexually transmitted infections are persistently endemic worldwide. Grouping countries, territories and areas according to SDG regions revealed that the prevalence and incidence of all four sexually transmitted infections, in both women and men, were highest in the Oceania Region. The small island states in this SDG region are part of the WHO Western Pacific Region, which is dominated by China (owing to its population size). Therefore, the levels of sexually transmitted infections and need for infection control in these island states are masked when viewing the estimates only by WHO Region. When using the World Bank classification of countries, the prevalence of gonorrhoea, trichomoniasis and syphilis were highest in low-income countries, territories and areas. The prevalence of chlamydia was highest in the upper middle-income countries, territories and areas, partly due to high estimates in some Latin American countries. Further research is needed to determine whether these estimates reflect methodological factors or differences in *C. trachomatis* transmission.

The 2016 estimates for chlamydia, gonorrhoea and trichomoniasis were based on a systematic review of the literature complemented by outreach to experts using the same methods as in 2012. The aim was to reduce bias and insure comprehensiveness in the search for data.^19^ For syphilis, the use of national estimates generated by a statistical model improves on the 2012 method by making use of historical trend data. The similarity between the published 2012 syphilis estimates and Spectrum STI generated estimates for 2012 provides reassurance about the validity of comparing the 2016 and 2012 estimates.

The study has limitations. First, limited prevalence data were available, despite an eight-year time window for data inclusion. Estimates for a given infection and region are therefore extrapolated from a small number of data points and ratios were used to generate estimates for some regions. For men, the lack of data was particularly striking. For syphilis, most data were from pregnant women, which might not reflect all women aged 15–49 years, or men. Second, the source studies include people in different age groups and used a range of diagnostic tests, so adjustment factors were applied to standardize measures across studies. Third, owing to the absence of empirical studies, incidence estimates were derived from the relationship between prevalence and duration of infection, and data on the average duration of infection for each of the four infections are also limited. Finally, because only studies among the general population were used, the prevalence and incidence in areas where key populations contribute disproportionately to sexually transmitted infection epidemics may have been underestimated despite the applied correction factor. These limitations have been discussed previously in detail.^6^

This study has implications for sexually transmitted infection programming and research. The quantity and quality of prevalence and incidence studies for sexually transmitted infections in representative samples of the general population, for both women and men, need improvement. Identifying opportunities to integrate data collection with clinical care platforms, such as HIV, adolescent, maternal, family planning and immunization is crucial. The recently developed WHO protocol for assessing the prevalence of sexually transmitted infections in antenatal care settings^153^ provides a framework and consistent methods that can be adapted for women and men. Comparing data across studies requires better understanding of the performance characteristics of diagnostic tests, and implications for estimates of the average duration of infection for each infection. The processes for producing future prevalence estimates could be made timelier and more efficient through continually updated systematic reviews,^154^ as well as technological solutions that automate searching of databases and facilitate high quality updates of reviews.

The global estimates of prevalence and incidence of four curable sexually transmitted infections are important in the broader global context, highlighting a continuing public health challenge. Prevalence and incidence data play an important role in the design and evaluation of programmes and interventions for sexually transmitted infections and in interpreting changes in HIV epidemiology. The global threat of antimicrobial resistance, particularly the emergence of *N. gonorrhoeae* resistance to the few remaining antimicrobials recommended for treatment, further highlights the importance of investing in monitoring prevalence and incidence.^155^ Estimates of prevalence and incidence are essential for calculations of the burden of disease due to sexually transmitted infections, which are needed to advocate for funding to support sexually transmitted infection programmes. These burden estimates can also be used to promote innovation for point-of-care diagnostics, new therapeutics, vaccines and microbicides. The WHO Global Health Sector Strategy on Sexually Transmitted Infections sets a target of 90% reductions in the incidence of gonorrhoea and of syphilis, globally, between 2018 and 2030.^9^ Major scale-ups of prevention, testing, treatment and partner services will be required to achieve these goals. The estimates generated in this paper, despite their limitations, provide an initial baseline for monitoring progress towards these ambitious targets.
